# Mitochondria: Key Organelle in Parkinson's Disease

**DOI:** 10.1155/2016/6230370

**Published:** 2016-08-07

**Authors:** Rubén Gómez-Sánchez, José M. Bravo-San Pedro, Matthew E. Gegg, Rosa A. González-Polo, José M. Fuentes

**Affiliations:** ^1^Department of Cell Biology, University of Groningen, University Medical Center Groningen, A. Deusinglaan 1, 9713 AV Groningen, Netherlands; ^2^Equipe 11 Labellisee par la Ligue Nationale contre le Cancer, Center de Recherche des Cordeliers, 75006 Paris, France; ^3^INSERM, U1138, 75006 Paris, France; ^4^Université Paris Descartes/Paris V, Sorbonne Paris Cité, 75006 Paris, France; ^5^Université Pierre et Marie Curie/Paris VI, 75006 Paris, France; ^6^Gustave Roussy Comprehensive Cancer Institute, 94805 Villejuif, France; ^7^Department of Clinical Neurosciences, UCL Institute of Neurology, Rowland Hill Street, London NW3 2PF, UK; ^8^Centro de Investigación Biomédica en Red sobre Enfermedades Neurodegenerativas (CIBERNED), Spain; ^9^Departamento de Bioquímica y Biología Molecular y Genética, Universidad de Extremadura, Facultad de Enfermería y Terapia Ocupacional, Avda de la Universidad S/N, 10003 Cáceres, Spain

Parkinson's disease (PD) is the second most common neurodegenerative disorder, characterized pathologically by loss of dopaminergic neurons in the* substantia nigra pars compacta*. The etiology of PD is still unknown, involving genetic and environmental factors; however mitochondrial dysfunction plays a central role in PD pathogenesis. In this regard, several PD-related proteins (PINK1, Parkin, DJ-1, LRRK2, and *α*-synuclein) are linked to mitochondrial function. Mitochondria are highly dynamic organelles involved in essential cellular functions, including energy production, calcium homeostasis, metabolism of amino acids and lipids, mtDNA replication, and programmed cell death. Moreover, mitochondrial homeostasis is tightly regulated by several pathways, including mitochondrial biogenesis, remodeling (fusion/fission), and clearance of damaged mitochondria by autophagy (mitophagy), among others. Mitochondrial dysfunction and the engagement of calcium channels during autonomous pacemaking have been implicated in the increased susceptibility of dopaminergic neurons to cell death in the* substantia nigra*.

This special issue is comprised of two reviews and five articles, which provide new insights into the molecular and cellular pathways related to mitochondria that may influence the pathogenesis of PD. 

In the first review (“Chaperone-Mediated Autophagy and Mitochondrial Homeostasis in Parkinson's Disease”), the authors summarize the current knowledge about autophagy and the relevance of this degradative pathway in the maintenance of mitochondrial function. Specifically, they highlight the link between mitochondrial dysfunction and impairment of chaperone-mediated autophagy activity in PD patients.

The second review, titled “Parkinson's Disease: The Mitochondria-Iron Link,” is focused on the relationship between accumulation of redox-active iron and the development/pathogenesis of PD. It is well-known that mitochondria are involved in the exchange of iron with the cytoplasm, with evidence suggesting that dysfunction in PD-related proteins (i.e., *α*-synuclein, Parkin, PINK1, DJ-1, LRRK2, and ATP13A2) leads to iron dysregulation. Because of the neurotoxicity linked to iron accumulation, Y. Muñoz et al. suggest that iron chelation is a potential therapeutic approach to slow down the progression of the disease.

Related to the previous review, the first research paper included in this special issue, entitled “Protection against Mitochondrial and Metal Toxicity Depends on Functional Lipid Binding Sites in ATP13A2,” examines the cytoprotective role of ATP132A and its consideration as a therapeutic target to reduce cellular toxicity. S. Martin et al. demonstrate that ATP132A requires the signaling lipids phosphatidic acid and phosphatidylinositol 3,5-bisphosphate to mediate protection to toxic Mn^2+^/Zn^2+^/Fe^3+^ concentrations and mitochondrial stress by the toxins rotenone and MPP^+^.

In the second research article, “Methyl-Arginine Profile of Brain from Aged PINK1-KO+A53T-SNCA Mice Suggests Altered Mitochondrial Biogenesis,” G. Auburger et al. use a powerful experimental model (PINK1-knockout with overexpression of A53T-SNCA double-mutant mice) to elucidate the polygenic etiology of PD. Based on quantitative global proteomics focused on methyl-arginine modifications, they report upregulation and downregulation of this specific posttranslational modification in several proteins, including some related to mitochondrial biogenesis such as CRTC1 and PSF. Moreover, posttranslational alterations of other identified factors could be required in molecular events linked to PD or other neurodegenerative disorders.

The third research article, “Altered Mitochondrial Respiration and Other Features of Mitochondrial Function in Parkin-Mutant Fibroblasts from Parkinson's Disease Patients” by W. Haylett et al., investigates mitochondrial health in* Parkin*-mutant fibroblasts from PD patients. Their results show that mitochondrial respiration and cell growth are higher in these cells, suggesting a compensatory mechanism in the absence of Parkin. Identification of this response could be a therapeutic target to preserve mitochondrial function in PD patients with Parkin mutations.

The fourth research paper of this special issue, “A Feed-Forward Circuit of Endogenous PGC-1*α* and Estrogen Related Receptor *α* Regulates the Neuronal Electron Transport Chain,” addresses the role of the key mitochondrial regulator PGC-1*α* in the activation of the nuclear-encoded mitochondrial electron transport chain (ETC) genes. R. Bakshi et al. show that PGC-1*α* regulates* ERRα* transcription. Interestingly, they report that pioglitazone treatment increases expression of endogenous* PGC-1α*,* ERRα*, and their ETC target genes. The modulation of the PGC-1*α* transcription network by drug administration could potentially be a clinical target for PD and other neurodegenerative diseases.

In the final review, “Activation Mechanism of LRRK2 and Its Cellular Functions in Parkinson's Disease,” the authors discuss the cellular role of LRRK2 and the recent research linking LRRK2-mediated PD to mitochondrial dysfunction and aberrant autophagy. In this regard, PD-associated mutations in* LRRK2* lead to impaired kinase and decreased GTPase activity. Thus, development of kinase inhibitors, as well as characterization of substrates and regulators of LRRK2, is essential in understanding LRRK2 pathogenesis and identifying potential targets for therapy.

The main purpose of this special issue is to shed light on the relevance of mitochondria as an essential organelle in postmitotic cells such as neurons and how mitochondrial damage contributes to the PD pathogenesis. An accurate and comprehensive understanding of mitochondrial quality control processes is critical to prevent cell death and development of age-related neurodegenerative disorders like PD. Current therapeutic strategies in PD are based on slowing down disease progression; however, they are not successful. New therapeutic approaches should be based on early biomarkers of PD and mitochondrial dysfunction is one promising target to be investigated.

*Rubén Gómez-Sánchez* *Rubén Gómez-Sánchez* *José M. Bravo-San Pedro* *José M. Bravo-San Pedro* *Matthew E. Gegg* *Matthew E. Gegg* *Rosa A. González-Polo* *Rosa A. González-Polo* *José M. Fuentes* *José M. Fuentes*

